# Isotretinoin Treatment for Acne Vulgaris: A Five-Year Retrospective Analysis of Clinical and Biochemical Adverse Effects

**DOI:** 10.3390/jcm14186473

**Published:** 2025-09-14

**Authors:** Igor Jarosław Feszak, Piotr Brzeziński, Sylwia Feszak, Aleksandra Kitowska, Monika Waśkow, Piotr Kawczak, Tomasz Bączek

**Affiliations:** 1 Institute of Health Sciences, Pomeranian University in Słupsk, 76-200 Słupsk, Poland; piotr.brzezinski@upsl.edu.pl (P.B.); kitowska.a@gmail.com (A.K.); monika.waskow@upsl.edu.pl (M.W.); or tomasz.baczek@upsl.edu.pl (T.B.); 2 Department of Pediatrics, Pediatric Oncology, and Immunology, Pomeranian Medical University, 71-252 Szczecin, Poland; sylmoraw@gmail.com; 3 Department of Pharmaceutical Chemistry, Faculty of Pharmacy, Medical University of Gdańsk, 80-416 Gdańsk, Poland; piotr.kawczak@gumed.edu.pl

**Keywords:** isotretinoin, retinoids, acne vulgaris, adverse effects, dyslipidemia

## Abstract

**Objectives:** Oral isotretinoin remains the most effective therapy for severe acne, but its exceptional efficacy is often accompanied by relatively frequent adverse effects. In this study, we quantified the frequency- and dose-related predictors of clinical and biochemical adverse effects during isotretinoin treatment in routine Polish practice. **Methods:** The records of 370 patients (mean age: 28 ± 12 years) who began isotretinoin treatment between June 2020 and June 2025 were reviewed. The mean daily isotretinoin and cumulative isotretinoin doses were 23.4 ± 9.1 mg and 88.3 ± 31.5 mg/kg, respectively. The adverse events documented at two-monthly visits were correlated with age and dosing. Lipid, hepatic, thyroid and prolactin panels were compared with age- and sex-matched controls using χ^2^ statistics and odds ratios (ORs). **Results:** Xerosis (70%), retinoid dermatitis (20%) and cheilitis (15.5%) predominated. Hand eczema rose with higher daily isotretinoin doses (ρ = 0.082; *p* = 0.037), whereas pruritus declined with greater cumulative isotretinoin exposure (ρ = −0.088; *p* = 0.037). Retinoid dermatitis was linked to a younger age (ρ = −0.080; *p* = 0.0286), whereas desquamation increased slightly with age (ρ = +0.083 *p* = 0.0228). Overall, dyslipidemia was twice as common as in the controls (OR: 2.06; 95% CI: 1.49–2.86; *p*-value: <0.0001), which was driven by an elevated total cholesterol (OR: 1.93; 95% CI: 1.34–2.77; *p*-value: 0.0004), LDL (OR: 3.40; 95% CI: 2.26–5.10; *p*-value: <0.0001) and triglycerides (OR: 1.95; 95% CI: 1.20–3.17; *p*-value: 0.0062) and decreased HDL (OR: 2.68; 95% CI: 1.75–4.10; *p*-value: <0.0001). Interestingly, hyperprolactinemia occurred eight-fold more often (OR: 8.42; 95%; 95% CI: 2.97–23.84; *p*-value: <0.00001). Aminotransferase and TSH elevations were infrequent and statistically non-significant. **Conclusions:** At moderate cumulative doses, isotretinoin was generally well tolerated; however, clinically relevant lipid and prolactin disturbances were frequent. Routine lipid and endocrine monitoring, early emollient prophylaxis and dose individualization are recommended to ensure safe isotretinoin usage in everyday practice.

## 1. Introduction

### 1.1. A Brief Historical Background of Isotretinoin

**Figure 1:**
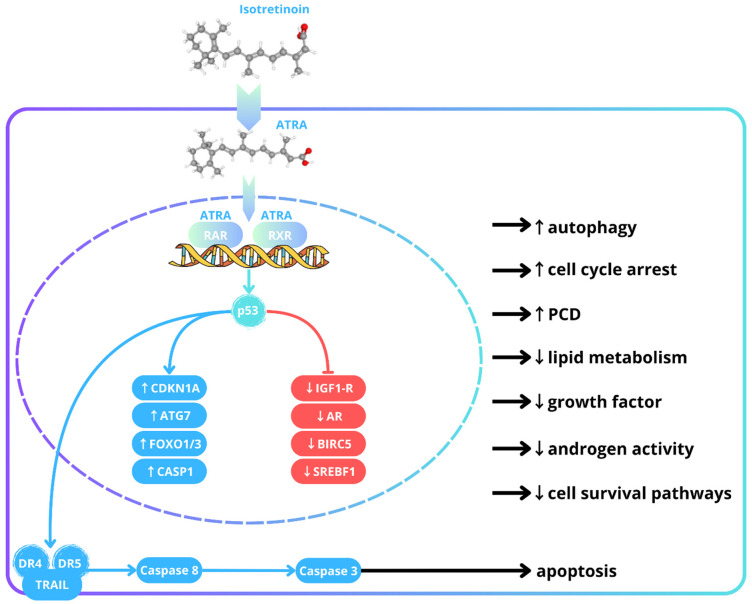
Brief overview of isotretinoin’s mechanisms of action based on the studies of [6,7,8]. ATRA—all-trans retinoic acid; RAR—retinoic acid receptor; RXR—retinoid X receptor; p53—tumor protein p53; ATG7—autophagy-related gene 7; CDKN1A—cyclin-dependent kinase inhibitor 1A; FOXO1/3—forkhead box O1/O3 protein; CASP1—caspase 1; PCD—programmed cell death; SREBF1—sterol regulatory element-binding transcription factor 1; IGF1—insulin-like growth factor 1 receptor; AR—androgen receptor; BIRC5—baculoviral inhibitor of apoptosis repeat-containing 5; TRAIL—TNF-related apoptosis-inducing ligand; DR4/5—death receptor 4/5.

Isotretinoin (ISO), also known as 13-cis-retinoic acid (Figure 1), is a synthetic derivative of vitamin A belonging to the first generation of retinoids [1]. Although first synthesized in 1955, its potential for treating psoriasis, genetic skin disorders, cystic acne and basal cell carcinoma was not explored until 1973 [2]. Finally, it was approved by the USA Food and Drug Administration (FDA) for the treatment of severe forms of acne in 1982, followed by Europe in 1983 [1,2].

### 1.2. A Brief Overview of Isotretinoin’s Mechanisms

After oral administration, isotretinoin (13-cis retinoic acid) is converted (isomerized) in cells into ATRA (all-trans retinoic acid), its biologically active form [3]. ATRA binds to the nuclear receptors retinoic acid receptor (RAR) and retinoid X receptor (RXR). This chemical complex binds to specific sequences in DNA, known as retinoic acid response elements (RAREs), and activates the transcription of target genes [4]. As a result, there is an increase in the expression of tumor protein p53 (p53), a tumor suppressor protein. ATRA, via p53, enhances the expression of genes responsible for cell cycle arrest—*cyclin-dependent kinase inhibitor 1A (CDKN1A)*; autophagy—*autophagy-related gene 7 (ATG7)*; and programmed cell death—*forkhead box O1 protein (FOXO1), forkhead box O3 protein (FOXO3),* and *caspase 1 (CASP1).* Concurrently, it downregulates genes linked to growth factor signaling—*insulin-like growth factor (IGF1)* and *insulin-like growth factor receptor (IGF1R*); androgen activity—*androgen receptor (AR)*; cell survival pathways—*baculoviral inhibitor of apoptosis repeat-containing 5 (BIRC5)*; and lipid metabolism—*sterol regulatory element-binding transcription factor 1 (SREBF1)* [3,4,5,6]. It is worth mentioning that p53 activates TNF-related apoptosis-inducing ligand (TRAIL) and its death receptors (DR4 and DR5). When TRAIL binds to DR4/DR5, it triggers the activation of caspases, such as caspase-8 and subsequently caspase-3, which leads to an apoptotic cascade in keratinocytes and sebocytes [5,6,7,8]. Ultimately, one of the key downstream effects of isotretinoin is its multifaceted action. It efficiently reduces sebaceous gland size and activity, resulting in an up to 90% decrease in sebum production. It inhibits keratinocyte proliferation in the granular layer, normalizes the stratum corneum, and regulates and stabilizes cell division in the spinous layer of the epidermis, thereby contributing to the restoration of balanced epidermal turnover. This description only provides a very brief overview of ISO’s complex mechanisms of action; these are presented in a diagram in Figure 1.

### 1.3. Dosing and Dose Optimization of Isotretinoin

The optimal dosage according to our current knowledge is 0.5 mg/kg of body weight/day for about the first 2–4 weeks, then a maintained dosage of 0.5–1 mg/kg of body weight/day for about 4–6 months [7,8]. The total (cumulative) isotretinoin dose should be 120–150 mg/kg of body weight; i.e., in the case of a person weighing about 60 kg, the total cumulative isotretinoin dose should be 7200–9000 mg during the course of treatment [5,6]. Recently, after an in-depth study, it was shown that a daily isotretinoin dose of 10–20 mg is also effective, leads to a similar final outcome and is associated with a lower intensity of adverse effects and an extended duration of therapy [9].

### 1.4. Clinical Applications of Isotretinoin

The primary indication for the use of ISO is severe acne vulgaris, with its effectiveness maintained in up to 70% of patients in long-term remission [10]. ISO is also used in other dermatological conditions, including rosacea [11], seborrheic dermatitis [12], hidradenitis suppurativa [13], lichen planus [14], dissecting cellulitis [15], ichthyosis [16,17], pityriasis rubra pilaris [18], psoriasis [19] and folliculitis decalvans [20]. It has also been applied in premalignant skin conditions and skin cancers such as keratoacanthoma [21] and squamous cell carcinoma [22]. Additionally, ISO has been used in certain lymphomas and leukemias, including acute promyelocytic leukemia as induction therapy combined with chemotherapy [23], juvenile chronic myelogenous leukemia as an adjunctive treatment [24], recurrent non-Hodgkin lymphoma as an adjuvant therapy [25], and mycosis fungoides/Sézary syndrome as part of systemic therapy [26,27]. Other than acne vulgaris, for which isotretinoin is the primary indication, the other conditions listed above should be treated with the first-line therapies recommended in the official guidelines specific to each disease. In the vast majority of these cases, isotretinoin is used as an adjunctive, second-line or off-label treatment with some degree of demonstrated efficacy in clinical studies. We present the main indication and various other applications of ISO in Table 1 below.

### 1.5. Adverse Effects of Isotretinoin

Despite its high efficacy and relatively broad spectrum of clinical applications, ISO is associated with a considerable number of adverse effects [9,28,29]. The most frequently observed side effects include xerosis (pathological dryness of the skin, usually manifesting with roughness, scaling and pruritus), dry lips, erythema, epistaxis, cheilitis, myalgias, pruritus, skin exfoliation, fatigue, headaches, arthralgias and retinoid dermatitis. Less commonly reported adverse effects comprise mood alterations, dry eyes, alopecia, abdominal discomfort, photosensitivity, seborrheic dermatitis, dryness of the oral mucosa, polydipsia and gastrointestinal disturbances [5,24,28]. Musculoskeletal discomfort and neuropsychiatric manifestations, including symptoms of depression and anxiety, have been documented as potential adverse effects of isotretinoin therapy [30,31]. Among these symptoms, teratogenicity remains the most critical risk. Isotretinoin is recognized as a potent teratogen, and its administration during pregnancy is known to cause irreversible congenital anomalies. The teratogenic effect of isotretinoin is primarily due to an excessive activation of RAR and RXR receptors in the developing embryo, which disrupts key processes in embryogenesis and impairs the proper differentiation of neural, mesenchymal and epithelial structures. Such interference can lead to profound, and often life-altering, fetal malformations [3,6]. Current estimates suggest that approximately 20–35% of fetuses exposed to ISO develop significant congenital malformations, while an additional 30–60% may exhibit other developmental impairments [32,33,34,35]. ISO may exert hepatotoxic effects, as evidenced by elevated serum aminotransferase levels [36]. It can also negatively impact lipid metabolism, resulting in increased levels of total cholesterol (TC), low-density lipoprotein (LDL) and triglycerides (TGs), along with a reduction in high-density lipoprotein (HDL) levels [37]. Furthermore, isotretinoin may impair thyroid function, which can be reflected in a decrease in serum concentrations of free tri-iodothyronine (fT3) and free thyroxine (fT4) [38,39]. Additionally, emerging evidence suggests that ISO administration may lead to a significant reduction in serum hormonal levels of luteinizing hormone (LH), prolactin (PRL), total testosterone, adrenocorticotropic hormone (ACTH), cortisol, insulin-like growth factor 1 (IGF-1) and growth hormone (GH) [39,40]. A brief summary of the adverse effects associated with isotretinoin therapy is presented in Table 2 below.

The aim of our study was to investigate the adverse effects of isotretinoin therapy in the Polish population.

## 2. Materials and Methods

This retrospective study included patients who began isotretinoin treatment for acne vulgaris in the period from June 2020 to June 2025. All patients underwent treatment at a single dermatology clinic by a board-certified dermatologist.

### 2.1. Inclusion Criteria

This study only included patients who had received a confirmed clinical diagnosis of acne vulgaris, were at least 15 years old at the beginning of therapy, began isotretinoin treatment during the specified study period and for whom follow-up data were available in the clinic records. Follow-up visits were scheduled approximately every two months, each involving a physical examination and clinical assessment by the treating dermatologist. Only patients with laboratory data obtained through the clinic’s affiliated laboratory were included, to ensure methodological consistency.

### 2.2. Exclusion Criteria

This study excluded patients who had isotretinoin contraindications, failed to attend follow-up appointments, lacked sufficient clinical documentation or whose data came from non-affiliated laboratories. This study also excluded patients who refused treatment and female patients who did not meet PPP requirements. It started with 513 patients, but 143 patients were excluded because of the abovementioned criteria.

### 2.3. Study Group Baseline Characteristics

The study group consisted of 370 isotretinoin-treated patients, with a sex distribution of 263 female (71.1%) and 107 male (28.9%) patients. Their mean age was 28.1 ± 12.2 years (range 15–74), and the age bands were as follows: 15–24 (196; 53.0%), 25–34 (85; 23.0%) and ≥35 (89; 24.1%). The mean height was 171.2 ± 7.8 cm, mean weight was 71.7 ± 14.9 kg, and the resulting mean BMI was 24.4 ± 4.4 kg/m^2^ (median 24.1; IQR 21.2–27.5). The mean daily isotretinoin dose was 23.4 ± 9.1 mg/day (range: 4.6–50; median 20.0; IQR 20.0–30.0), and the mean cumulative dose was 88.3 ± 31.5 mg/kg (range: 40.0–144.7; median 80.0; IQR 50.0–120.0). The baseline characteristics of the study group are summarized in Table 3.

### 2.4. Control Group Selection, Matching and Baseline Characteristics

The control cohort was derived from the database of the same clinic within the same time window as the isotretinoin-treated patients, ensuring comparability of the source population. In particular, we included outpatients with complete demographic data and laboratory records, aged 15–71 years, and without any history of isotretinoin or systemic retinoid use. Patients with conditions likely to bias biochemical or endocrine outcomes (e.g., pregnancy, pituitary adenoma, severe chronic liver or renal disease or active malignancy) were excluded. Each control was assigned an index date (defined as the first eligible outpatient encounter), and duplicate records were removed. Although the eligibility window covered ages 15–71, the final assembled control group comprised patients between 20 and 40 years of age, reflecting the real-world distribution of available records. After exclusion, 300 patients were finally included as controls.

To maximize comparability, controls were matched to treated patients using a structured two-step algorithm. First, exact matching was performed according to sex and age band (15–24, 25–34, ≥35). Second, within each stratum, nearest-neighbor matching without replacement on chronological age was applied using a caliper of ±2 years. This approach ensured close alignment while retaining a sufficient sample size. As a perfect proportional alignment across bands was not always achievable, minor imbalances remained; therefore, the main analyses compared the full treated and control cohorts with multivariable adjustment, while the matched subset was analyzed in sensitivity analyses. Anthropometric variables (height, weight, BMI) were collected and reported descriptively in baseline tables but were not used in the matching algorithm.

The control group consisted of 300 patients, with a sex distribution of 204 female (68.0%) and 96 male (32.0%) patients. Their mean age was 29.44 ± 6.31 years (range 20–40), and the age bands were as follows: 15–24 (85; 28.3%), 25–34 (130; 43.3%) and ≥35 (85; 28.3%). The mean height was 176.0 ± 9.5 cm, mean weight was 70.5 ± 13.8 kg, and the mean BMI was 22.7 ± 3.9 kg/m^2^ (median 22.5; IQR 20.5–24.9). The baseline characteristics of the control group are summarized in Table 4.

### 2.5. Treatment Protocol

The treatment started only after a detailed consultation with the patients. Patients received both the manufacturer’s summary of product characteristics (SmPC) and a brochure about isotretinoin therapy, along with sample skincare products. Patients with contraindications to ISO were offered alternative treatment options, as determined by the dermatologist. Female patients were required to enroll in the European Pregnancy Prevention Program (PPP) prior to therapy [41,42]. They received verbal and written counseling regarding the teratogenic risks and were instructed to use either one highly effective method or two complementary contraceptive methods, starting at least one month before therapy and continuing throughout treatment. A pregnancy test was performed within three days prior to the first dose, in line with PPP guidelines. All female patients underwent a gynecological consultation to exclude early pregnancy, due to the possibility of false-negative test results. Treatment began with a dose ranging from 20 mg to 30 mg per day. The dose was adjusted after 1–2 months, with the goal of achieving a dose of 0.5 mg/kg/day. The target cumulative isotretinoin dose was 120–150 mg/kg, in accordance with established therapeutic guidelines. In cases of poor tolerability, such as mucocutaneous side effects, elevated liver enzymes, or dyslipidemia, the dosing was adjusted. Some patients were temporarily switched to an alternative dosing regimen (0.5–1 mg/kg every other day) until their side effects resolved. All modifications were made at the discretion of the treating dermatologist, based on clinical and laboratory findings.

### 2.6. Data Analysis and Ethical Considerations

The assessment of adverse effects relied on patient histories, combined with clinical examination results and laboratory test outcomes. The documented adverse effects were categorized into the following groups: skin changes, oral changes, nasopharyngeal changes, ophthalmic changes, musculoskeletal changes, gastrointestinal changes, infections, mood and neurological changes and others. To assess relationships between patient age, mean daily isotretinoin dose, cumulative isotretinoin dose and the occurrence of specific adverse events, a Spearman rank correlation test was used as a non-parametric method for evaluating the strength and direction of monotonic associations between variables. Results are presented as Spearman correlation coefficients with corresponding *p*-values, with *p*-values < 0.05 considered statistically significant. The biochemical parameters analyzed included TC, LDL, HDL, TG, aminotransferase, TSH and PRL levels. Odds ratios (ORs) with 95% confidence intervals (CIs) were calculated for each parameter. Statistical significance was assessed using a chi-square test. Missing data were excluded from the analysis. All statistical analyses were performed using R software (version 4.3.2; R Foundation for Statistical Computing, Vienna, Austria). This study was conducted in accordance with the Declaration of Helsinki and was approved by the Bioethics Committee at the Regional Medical Chamber in Gdańsk (approval number: KB-19/24 of 18 June 2024). All data were collected and analyzed in a fully anonymized form, preventing the identification of individual patients.

## 3. Results

### 3.1. Adverse Effects of Clinical Assessment

The results of reported adverse effects are presented below in Table 5A–I.

### 3.2. Correlations Between Adverse Effects and Clinical Variables

**Figure 2:**
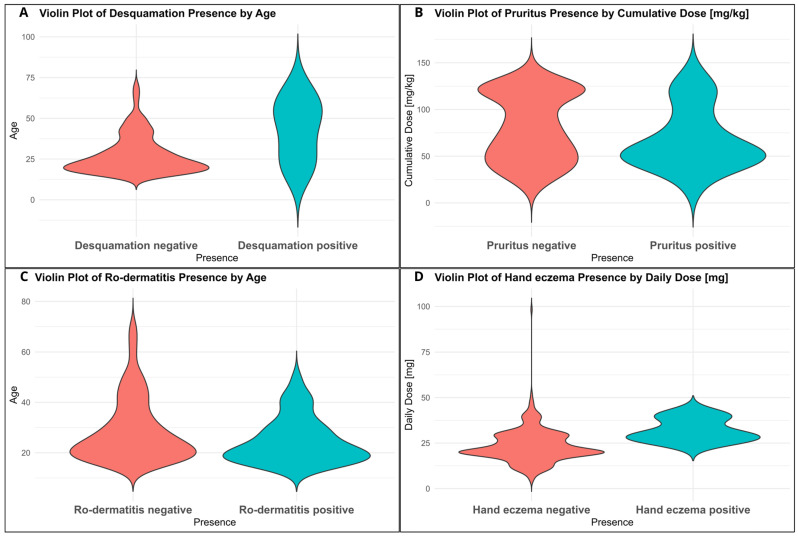
(**A**–**D**) Violin plots of correlations between adverse effects and clinical variables (created in R software version 4.3.2).

In this cohort, hand eczema showed a positive correlation with daily isotretinoin dose (ρ = +0.082, *p* = 0.037), indicating that higher daily isotretinoin doses were associated with a slightly increased occurrence of this adverse effect. The research data showed that patients who received higher total cumulative isotretinoin doses experienced pruritus less frequently (ρ = −0.088, *p* = 0.037). Regarding age-related associations, retinoid dermatitis (ro-dermatitis) showed a negative correlation with age (ρ = −0.080, *p* = 0.029), implying that this adverse effect tended to occur more frequently in younger patients. In contrast, desquamation displayed positive correlations with age (ρ = +0.083, *p* = 0.023), indicating a marginally higher frequency of this effect among older individuals. Overall, these correlations were statistically significant but weak in magnitude, suggesting that while dose and age may influence the occurrence of certain adverse effects, their clinical impact in isolation is likely limited. We have summarized these results in Table 6 and Figure 2A–D.

### 3.3. Impact of Isotretinoin on Lipid Profile

Table 7 below presents descriptive statistics for total cholesterol, HDL, LDL and triglycerides in patients treated with isotretinoin.

Data analysis revealed an association between ISO and lipid profile deviations outside the normal range. In the study group receiving ISO treatment, TC (OR: 1.93; 95% CI: 1.34–2.77; *p* = 0.0004) and TG (OR: 1.95; 95% CI: 1.20–3.17; *p* = 0.0062) levels above the reference range were almost twice as frequent compared to the age–gender-matched control group. LDL levels above the normal range were more than three times as frequent (OR: 3.4; 95% CI: 2.26–5.10; *p* < 0.0001). On the other hand, HDL levels below the reference range were more than twice as frequent as in the control group. We took into account how often any of the above parameters caused dyslipidemia in the diagnosis and calculated that it was more than twice as frequent in the ISO group (OR: 2.06; 95% CI: 1.49–2.86; *p* < 0.0001). Results of this analysis are shown in Table 8 below.

**Figure 3:**
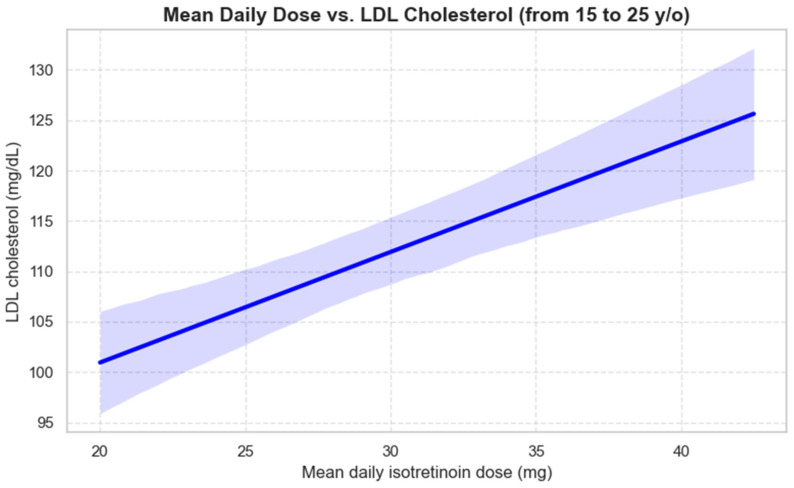
Linear regression of mean daily isotretinoin dose vs. LDL cholesterol in patients aged between 15 and 25 years (created with Python software version 3.13); Blue band = 95% confidence interval—it shows the uncertainty of the estimated mean LDL at each dose of ISO.

This study shows that patients aged 15–25 years who received isotretinoin had a statistically significant positive correlation between their daily isotretinoin dose and LDL levels. The results indicate that each daily increase of 1 milligram of ISO resulted in an average LDL level increase of 1.11 mg/dL (r = 0.28, *p* = 0.030). The actual LDL change may differ depending on various patient-specific factors. The results indicate that higher doses of isotretinoin treatment may lead to lipid profile changes through elevated LDL cholesterol levels. The linear regression plot showing this relationship appears in Figure 3 below.

### 3.4. Isotretinoin on Aminotransferases

The serum AST levels in the study group ranged from 5.3 to 65.0 U/L, with a mean of 20.8 U/L (SD = 7.3) and a median of 20.0 U/L. ALT levels ranged from 3.17 to 79.0 U/L, with a mean of 18.2 U/L (SD = 10.6) and a median of 15.75 U/L. The analysis of aminotransferase data did not demonstrate significant results. Although the incidence of elevated AST and ALT in the ISO group was more than 2 and 1.5 times higher, respectively, than in the control group, statistical significance was not achieved. Further information on this topic is provided in Table 9 below.

**Figure 4:**
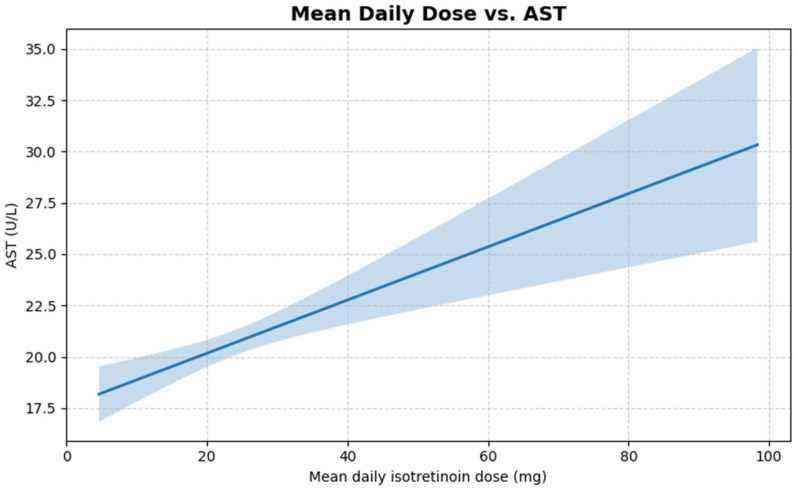
Linear regression of mean daily ISO dose vs. AST (created with Python software version 3.13); Blue band = 95% confidence interval—it shows the uncertainty of the estimated mean AST at each dose of ISO.

We found that there was a positive Pearson correlation between the mean daily isotretinoin dose and AST (*r* = 0.14; 95% CI = 0.02–0.25; *p* = 0.023), indicating that higher day-to-day dosing was accompanied by a modest rise in AST values. Although the effect size is weak, its significance suggests a dose-responsive correlation with aminotransferase elevation within the treated cohort. A linear regression plot showing this relationship is shown in Figure 4 below.

### 3.5. Isotretinoin on Thyroid-Stimulating Hormone

The TSH levels in the study group ranged from 0.005 to 119.72 µIU/mL, with a mean of 2.76 µIU/mL (SD = 8.18). The median value was 1.77 µIU/mL. The odds of having an elevated TSH level in patients taking ISO in the study group were approximately 1.5 times higher than in the control group. However, this result is not statistically significant. Table 10 below shows the data of this analysis.

### 3.6. Impact of Isotretinoin on Prolactin

The mean prolactin level was 20.6 ng/mL (SD = 10.0 ng/mL); values ranged from a minimum of 2.05 ng/mL to a maximum of 55.22 ng/mL. The median value was 18.77 ng/mL. Only two patients in the study group had prolactin levels below the reference range, while thirty-nine had levels above it. Compared to the control group, individuals receiving isotretinoin were over eight times more likely to exhibit elevated prolactin levels; this result was statistically significant (Table 11).

### 3.7. Timing of Adverse Effects

In our cohort, the earliest and most frequent signs of intolerance were mucocutaneous. Cheilitis was the most frequent intermediary manifestation preceding initial xerosis, which appeared on average within the first week after initiation of therapy. The peak of reported dryness occurred in the fourth week. Epistaxis was consistently reported by the end of the first month of therapy and persisted until the third month. Laboratory lipid abnormalities were usually apparent by the fourth week. Musculoskeletal complaints emerged later, with a median onset around three months. Overall, these time frames establish a temporal association between isotretinoin exposure and adverse effects—a key component of causality according to the Naranjo criteria [43]. More detailed information on the expected onset of the most frequent adverse effects is summarized in Table 12.

## 4. Discussion

### 4.1. Optimizing Isotretinoin Dosing: Evidence for Low-Dose Efficacy

The medical field has adopted the use of low-dose and very-low-dose isotretinoin treatment protocols during the past ten years, which provide effective results while minimizing adverse effects; however, this comes at the expense of a longer treatment duration and a potentially higher risk of relapse after treatment’s discontinuation. A randomized study conducted by Rademaker et al. (2013) showed that adults with chronic low-activity acne who took 5 mg of ISO per day experienced a reduced number of lesions and improved quality of life with few negative side effects [44,45]. The effectiveness of 20 mg per day and 20 mg every other day for treating moderate to severe acne has been established in a prospective and randomized study conducted by Rasi et al. (2024) [46]. The treatment methods proved to be safe for patients, although the daily treatment schedule provided better results for patients with more serious conditions [46]. Meta-analyses and systematic reviews summarizing various regimens (including 10–20 mg/day) have indicated that lower doses may be particularly useful in adults and in patients who are prone to adverse effects [47,48]. In these scenarios, the relapse rate depends on the total cumulative dose and duration of treatment [47,48]. From a practical perspective, a lower dose (10 or 20 mg per day) is considered an acceptable choice if the patient prefers a milder safety profile and the physician accepts a longer course and the need for a closer monitoring of effects.

### 4.2. Clinical Adverse Effects

The analysis of our retrospective study revealed that the most common adverse effects in our cohort were mucocutaneous, i.e., xerosis (70%), retinoid dermatitis (20%), cheilitis (15.5%), xerophthalmia (4.3%) and epistaxis (3.7%). Mood-related adverse effects were minimal, with only two patients having experienced the worsening of pre-existing depression. The frequency of these adverse events was similar or even lower in our study compared to that of most previous studies on this topic. Interestingly, the overall frequency of most side effects appeared to be lower in our analysis than that in most previous studies. For instance, Brzeziński et al. (2017) conducted a large retrospective analysis of over 3000 patients from Poland and Hungary and found that xerosis affected 94% of patients, and epistaxis occurred in 47.26% of cases, while cheilitis affected 41% [29]. According to the aforementioned study, the incidence of retinoid dermatitis was estimated as 11% of all cases [29]. The study by Rademaker et al. (2010) examined 1743  patients in New Zealand. Their cohort experienced cheilitis in 78% and epistaxis in 38% of cases, respectively, significantly lower than the epistaxis and cheilitis rates reported in our study [9]. The study by Rademaker also documented low psychiatric side effects, since only 13 patients stopped their treatment due to mood symptoms, and there were no documented cases of suicidal behavior, similar to our study. According to Kapała et al. (2022), who conducted a meta-analysis of 15 isotretinoin studies, the combined evidence demonstrated that xerosis affected 49% of patients, cheilitis affected 41% of patients, and skin irritation symptoms related to retinoid dermatitis affected 27% of patients [28]. The study by Straus et al. (2001) evaluated the prevalence of cheilitis  in 90.3% of conventional isotretinoin patients and 91.3% of patients receiving micronized isotretinoin in a multicenter USA randomized controlled trial. The study documented xerosis in 53% of patients and epistaxis in about 35–45% of cases, depending on the dosage regimen [49]. According to the Beck Depression Inventory-II formal assessment scale, none of the patients developed a clinically important depression in their study. The relatively low frequency of side effects observed in our study may reflect a combination of favorable treatment conditions and a degree of underestimation. In our study, patients received a lower-than-standard cumulative isotretinoin dose of approximately 88 mg/kg, alongside a modest average daily isotretinoin dose of 23.4 mg/day, which likely contributed to our better tolerability. On the other hand, it is possible that some factors artificially lowered our recorded rates. Patients may have under-reported mild symptoms that they perceived as unimportant, and as a retrospective analysis, some data might not have been captured consistently in documentation. These possibilities should be considered when comparing our results to the higher side effect rates reported in broader studies.

### 4.3. Correlation Between Adverse Effects, Age and Dose

Our analysis revealed four modest, yet statistically significant, connections between adverse events and patient variables (|ρ| ≈ 0.08). Although subtle, these signals gain meaning when set beside earlier studies. Daily isotretinoin dose escalation coincided with a higher frequency of hand eczema in our patients (ρ = +0.082). The study by Bagatin et al. (2020) supports this discovery, because they established that dryness-related skin changes directly depend on dosage levels [50]. In our study, a cumulative isotretinoin dose increase led to a reduction in pruritus symptoms (ρ = −0.088), and the incidence of retinoid dermatitis appeared more frequently among younger patients (ρ = −0.080). The study by Chu et al. on patients treated for cutaneous T-cell lymphoma with ISO (CTCL)  shows that patients with high ISO doses (≈2 mg/kg/day) are required to reduce their dosing when dermatitis becomes severe [51]. Our age signal implies that suppressing sebum production too aggressively may trigger a stronger skin reaction in adolescents, highlighting the need for dosing studies that analyze age groups separately. Finally, we noted a mild rise in desquamation with advancing age (ρ = +0.083). The study by Bagatin et al. demonstrates that older patients primarily experience dryness  on their skin, which supports the theory that skin barrier weakness increases with age [50]. To sum up, the initial use of low doses, together with ceramide-based moisturizers, at the beginning of treatment appears to be the most suitable approach for patients. On the other hand, given the observational design of this study, the absence of adjustment for sex, BMI and atopic status and the small correlation coefficients we observed, it is quite likely that other, unmeasured variables influence these associations. Therefore, future studies should aim to identify and characterize these additional factors.

### 4.4. Lipid Profile

In our study, TC and TGs exceeded reference limits at nearly double the odds rate, while LDL levels increased more than three times as often and low HDL occurred twice as often in patients receiving isotretinoin compared to matched controls. The lipid profile disturbances observed in our study population matched the results that have been reported in studies conducted worldwide [37,51,52,53]. According to Krishna et al. (2025) in their worldwide review, isotretinoin treatment increases TC, TGs and LDL while decreasing HDL [53]. A study by Alrasheed et al. (2024) in Saudi Arabia showed the same changes at an average isotretinoin dose of 27 mg/day [51]. The study by Nejad et al. (2024) demonstrated that, even with a daily isotretinoin dose of 20 mg, LDL levels increased, while HDL levels remained mostly stable [52]. Together, these studies confirm that our odds ratio patterns reflect a universal response, rather than a region-specific anomaly. Several features add weight to our findings. The age- and sex-matched control group of nearly equal size enabled us to establish that lipid changes were caused by isotretinoin rather than initial population differences. The measurement of all four lipid fractions (TC, LDL, HDL, TGs) in the same certified laboratory provided a comprehensive view of dyslipidemia and maintained analytical consistency. We presented odds ratios with 95% confidence intervals for every fraction, giving clinicians an intuitive gauge of risk rather than simple mean shifts. This study did not include records of diet or exercise, or concomitant medications, which independently affect serum lipid levels. The presence of missing values decreased the number of participants available for analysis of HDL and LDL fractions, thus potentially increasing the width of confidence intervals. A future multicenter prospective study including lipid testing and lifestyle monitoring would enhance the understanding of lipid disorders during ISO treatment.

### 4.5. Aminotransferases

In our cohort, the proportion of patients whose AST or ALT levels exceeded the laboratory’s upper limit was modestly higher after ISO treatment than for untreated controls (AST: 5.5% vs. 2.5%; ALT: 6.5% vs. 4.0%). Still, the odds ratios failed to reach statistical significance. These figures mirror the real-world data of Vieira et al., who observed asymptomatic elevations in 8–9% of 70 adolescents, none requiring ISO withdrawal [54]. They also align with the meta-analysis by Lee et al. (26 trials, n = 1574), where AST and ALT remained well below the high-risk threshold, and clinically relevant hepatotoxicity occurred in <3% of cases [55]. These data are not enough to connect hepatotoxicity with ISO treatment in the case of our study. On the other hand, in our analysis, a weak but significant correlation between mean daily isotretinoin dose and AST (r = 0.14, *p* = 0.023) is observed. This may suggest that higher day-to-day exposure mildly stresses hepatocytes. Evidence for this remains conflicting; a large cohort of Saudi Arabian patients reported overall AST/ALT increases but found no association with the prescribed dose [51]. On the other hand, a review of high-dose courses (Blasiak et al., 2023) only showed clinically relevant AST spikes when cumulative isotretinoin exposure exceeded ≈220 mg/kg [56,57]. Our data fall between these extremes and indicate that a high daily isotretinoin dose, even over a short course, may place a greater acute burden on hepatocytes than the same total dose administered more gradually.

### 4.6. Thyroid-Stimulating Hormone

In our study, the risk of an above-reference TSH value was 1.5-fold higher in isotretinoin recipients than in controls, although this result was not statistically significant. On this, two prospective series have shown clear, statistically significant increases: Uyar et al. (2016) noted TSH rising from 1.6 ± 0.7 to 2.3 ± 0.9 µIU/mL over six months, with concomitant declines in free T3 and T4 [58]. Yıldırım et al. (2017) reported a similar increase—from 1.6 to 2.3 µIU/mL—in 51 patients followed up for the same duration [59]. The shortcomings of this part of the study include that the modest sample size produced wide confidence intervals, so a lack of statistical significance may simply reflect inadequate power rather than a true absence of effect. The right-skew of TSH values inflated variance and further reduced the sensitivity of parametric tests to detect subtle treatment-related changes. Finally, because free T4 and free T3 were not measured, we could not compute compensatory indices, such as the T4-to-TSH ratio or explore peripheral T4 → T3 conversion, making it difficult to determine the thyroid toxicity of ISO with certainty.

### 4.7. Prolactin

This study revealed that patients who received ISO treatment had PRL levels above referral ranges eight times more often than control group patients, with an odds ratio of 8.42 (95% CI = 2.97–23.84; *p*-value < 0.00001). Data regarding the effects of ISO on the hypothalamic–pituitary–peripheral axis are heterogeneous, but most studies have indicated a tendency towards a decrease in pituitary and peripheral hormone concentrations. In the studies of Feily et al. (2019) and Karadağ et al. (2015), PRL levels decreased by approximately 35% and 25%, respectively, after 3 months of low-dose therapy (*p*-values of 0.001 and <0.0001, respectively) [39,60]. Significant reductions in LH, PRL, total testosterone, ACTH, IGF-1 and GH were also observed in those studies, and the effects were more pronounced at higher daily doses [40,55]. The only known exception to this pattern is a case report of macroprolactinemia triggered by isotretinoin immuno-interference, which produced a spurious elevation on routine immunoassay (Siddiqi et al., 2021) with a PRL level of 2055 mIU/L. Several factors could explain our discordant results [61]. Our sample included both sexes and a wider age range, whereas most earlier studies focused on young women, in whom isotretinoin may enhance dopaminergic D2 receptor tone and suppress lactotroph output. Assay interference by macroprolactin cannot be excluded, as polyethylene glycol precipitation was not routinely performed. From a clinical perspective, routine PRL testing during ISO therapy is not recommended. The current guidelines in the *Journal of the American Medical Association* (JAMA) and the *Journal of the American Academy of Dermatology* (JAAD) do not include PRL as a required measurement for ISO therapy [1,55]. The required standard tests include lipid profile and aminotransferases, while monthly contraceptive surveillance and pregnancy testing according to pregnancy prevention programs (PPP/iPLEDGE) are mandatory for women of reproductive age [1,55]. In our opinion, PRL testing should be considered in patients with menstrual irregularities, galactorrhea, headaches or visual disturbances or in men with gynecomastia or hypogonadism. A macroprolactin test should be performed to verify any abnormal results, as it helps to prevent unnecessary overdiagnosis.

### 4.8. Study Limitations

This study utilized a retrospective design to analyze numerous real-world patient cases treated in routine practice. However, this design restricted researchers from controlling the consistency and completeness of the collected data. This study, conducted at a single clinic, provided consistent treatment and follow-up care from an experienced dermatologist, but this approach might decrease the applicability of the results to different clinical environments and patient groups. The manual data extraction process from paper and electronic records enabled researchers to conduct thorough individual case assessments; on the other hand, the manual data extraction process exposed this study to human errors, including both incorrect interpretations and missing important details. Together with the patient-reported symptoms, the clinical assessments offered a useful and patient-focused view of treatment tolerability; however, the reporting of mild or transient adverse effects might have been insufficient. Additionally, patient interviews could be influenced by personal subjective biases, because they rely on individual perceptions.

It is also important to recognize the absence of prospective data. Since it was a retrospective analysis, the results reveal associations rather than relationships. A prospective, multicenter study would be required to confirm the temporal and mechanistic links suggested by our findings. Moreover, the lack of long-term follow-up after treatment’s completion prevented assessment of relapse rates, delayed adverse effects or sustained metabolic and endocrine alterations. This study was also limited by availability of laboratory data. Certain biochemical parameters, including PRL and individual lipid fractions, were assayed only in a subset of patients. This partial coverage could decrease the statistical power for some analyses and potentially the robustness for the findings for subgroups.

Furthermore, parameters of lifestyle and metabolic data were not obtained regularly. Data on changes in BMI, physical activity, dietary intakes, alcohol or smoking status were not accessible. These factors are known to affect lipid and endocrine parameters and could have contributed to the observed variability. Lastly, the demographics of the cohort need to be kept in consideration. Over-representation among young patients, as well as women, might have influenced the incidence as well as the degree of adverse effects. Therefore, the applicability of these results to older populations, men or patients with significant comorbidities may be limited. In addition, the sample size, although relatively large, was insufficient to evaluate very rare adverse events, particularly those related to inflammatory bowel diseases (IBD), such as Crohn’s disease and *colitis ulcerosa*.

The above limitations should be taken into account when analyzing this study’s results. Despite these limitations, we believe that our study still offers a meaningful contribution to the understanding of isotretinoin’s safety in real-world clinical practice and may serve as a useful basis for future research.

### 4.9. From the Practical Point of View

We recommend the following testing pattern in clinical practice [62,63]: measuring the lipid profile and ALT/AST before starting treatment and, in women of reproductive age, performing a PPP/iPLEDGE-compliant pregnancy test. This should be followed by early monitoring during the first two months after initiation or when reaching the peak dose, as this is when lipid profile abnormalities most often become apparent. If the results are optimal or the patient is asymptomatic, further testing should be performed in the fifth–sixth month of treatment, with more frequent monitoring in those with risk factors (high baseline dyslipidemia, obesity, concomitant liver disease, inflammatory bowel disease). Subsequent monitoring should be performed every 6 months. It is important to remember that severe abnormalities—such as significant hypertriglyceridemia or pronounced hepatotoxicity—are rare but require a rapid response, including possible dose modification or immediate discontinuation of medication. Therefore, decisions should be made on an individualized basis, and, if deviations occur, the monitoring frequency should be increased. Regarding PRL, testing should be performed in the presence of clinical symptoms. If the PRL level is elevated, it should be confirmed via a macroprolactin (PEG) test due to the risk of falsely elevated results. It should also be noted that, according to some studies, ISO may induce the onset IBD. Therefore, we recommend particular diligence during the patient’s physical examination and, if such a course is suspected, consider expanding the evaluation to include a complete blood count (CBC), serum C-reactive protein (CRP), calprotectin (FC) and colonoscopy [64,65].

## 5. Conclusions

This retrospective study of isotretinoin-treated patients with acne vulgaris revealed multiple important clinical findings regarding the drug’s adverse effects. The observed mucocutaneous side effects were the most common clinical effects, yet their frequencies remained lower than those documented in similar large-scale studies. The moderate daily isotretinoin dosing at 23.4 mg/day, combined with the reduced cumulative isotretinoin dose of 88.3 mg/kg, probably led to better tolerance in patients. Statistically important relationships between specific adverse effects and patient-specific variables were revealed, and although retinoid dermatitis occurred more frequently in younger patients, desquamation appeared slightly more often in older patients. A weak relationship was identified between hand eczema’s development and a higher daily isotretinoin dose, while pruritus symptoms decreased with an increasing cumulative dose exposure. These associations match existing research findings, which supports the practice of individualized dose selection. The biochemical results from our study demonstrate that isotretinoin affects lipid profiles in patients. Our results demonstrated a significant elevation of dyslipidemia odds in patients, which matches previous research findings. The AST levels showed a weak positive correlation with the daily isotretinoin dose, although the changes did not reach statistical significance. The results indicate that the liver may respond to isotretinoin through a dose-dependent mechanism. The findings regarding endocrine outcomes were particularly significant, because patients developed elevated prolactin levels at a rate more than eight times higher than the expected findings from previous studies. These unexpected findings demonstrate the requirement for additional studies on the hormonal effects of isotretinoin across different population groups and treatment protocols. The effectiveness of isotretinoin for treating severe acne remains high, but our findings demonstrate the need for individualized risk assessments, continuous laboratory tests and careful dosing strategies because of its effects on lipids and hormones. The validation of these associations and the development of safer personalized isotretinoin therapy guidelines will require future prospective multicenter studies.

## Figures and Tables

**Table 1:** Applications of isotretinoin based on the studies of [10,11,12,13,14,15,16,17,18,19,20,21,22,23,24,25,26,27].

Applications of Isotretinoin			
**Main Application**	Other Dermatological Applications	Cancers	Leukemias/Lymphomas
Severe acne	Rosacea Seborrheic dermatitis Hidradenitis suppurativa Lichen planus Dissecting cellulitis Ichthyosis Pityriasis rubra pilaris Psoriasis Folliculitis decalvans	Keratoacanthoma (KA) Squamous cell carcinoma (SCC)	Acute promyelocytic leukemia (APL) Juvenile chronic myelogenous leukemia (CML) Recurrent non-Hodgkin lymphoma (NHL) Mycosis fungoides/Sézary syndrome (MF/SS)

**Table 2:** Brief summary of adverse effects of isotretinoin based on the studies of [26,27,28,29,30,31,32,33,34,35,36,37,38,39].

Adverse Effects of Isotretinoin		
**Common**	Less Common	Biomarkers and Hormones
Xerosis Erythema Epistaxis Cheilitis Myalgias Pruritus (itching) Skin exfoliation Arthralgias Retinoid dermatitis	Mood alterations Depression exacerbation Dry eyes Photosensitivity Seborrheic dermatitis Dry dandruff Xerophthalmia Polydipsia Abdominal pain	Liver: ↑ AST, ↑ ALT, ↑ GGTP, ↑ ALP Lipid profile: ↑ TC, ↑ LDL, ↑ TGs, ↓ HDL Thyroid: ↓ fT3, ↓ fT4, ⇅ TSH. Pituitary: ↓ LH, ↓ PRL, ↓ ACTH, ↓ GH, Peripheral: ↓ testosterone, ↓ cortisol, ↓ IGF-1

ALT/AST—alanine/aspartate aminotransferase; LDL—low-density lipoprotein; HDL—high-density lipoprotein; TSH—thyroid-stimulating hormone; fT3—free triiodothyronine; fT4—free thyroxine; LH—luteinizing hormone; PRL—prolactin; ACTH—adrenocorticotropic hormone; IGF-1—insulin-like growth factor-1; GH—growth hormone; ↑—increase above the upper limit; ↓— decrease below the lower limit.

**Table 3:** Baseline characteristics of isotretinoin-treated patients (n = 370).

Variable	Value
Age	Mean: 28.1; SD: 12.2; Median: 24; IQR: 19–34
Sex	Female: 263 (71.1%); Male: 107 (28.9%)
Age bands	15–24: 196 (53.0%); 25–34: 85 (23.0%); ≥35: 89 (24.1%)
Height (cm)	Mean: 171.2; SD: 7.8; Median: 171; IQR: 166–177
Weight (kg)	Mean: 71.7; SD: 14.9; Median: 70.0; IQR: 61.2–81.6
BMI (kg/m^2^)	Mean: 24.4; SD: 4.4; Median: 24.1; IQR: 21.2–27.5
Daily dose (mg/day)	Mean: 23.4; SD: 9.1; Median: 20.0; IQR: 20.0–30.0
Cumulative dose (mg/kg)	Mean: 88.3; SD: 31.5; Median: 80.0; IQR: 50.0–120.0

SD—standard deviation; IQR—interquartile range; BMI—body mass index.

**Table 4:** Baseline characteristics of control patients (n = 300).

Variable	Value
Age	Mean: 29.44; SD: 6.31; Median: 29; IQR: 24–35
Sex	Female: 204 (68.0%); Male: 96 (32.0%)
Age bands	15–24: 85 (28.3%); 25–34: 130 (43.3%); ≥35: 85 (28.3%)
Height (cm)	Mean: 176.0; SD: 9.5; Median: 176; IQR: 170–182
Weight (kg)	Mean: 70.5; SD: 13.8; Median: 69.0; IQR: 61.0–78.0
BMI (kg/m^2^)	Mean: 22.7; SD: 3.9; Median: 22.5; IQR: 20.5–24.9

SD—standard deviation; IQR—interquartile range; BMI—body mass index.

**Table 5:** (**A**) Isotretinoin therapy adverse effects—skin changes, n = 370. (**B**) Isotretinoin therapy adverse effects—oral changes, n = 370. (**C**) Isotretinoin therapy adverse effects—nasopharyngeal changes, n = 370. (**D**) Isotretinoin therapy adverse effects results—ophthalmic changes, n = 370. (**E**) Isotretinoin therapy adverse effects—musculoskeletal changes, n = 370. (**F**) Isotretinoin therapy adverse effects—gastrointestinal changes, n = 370. (**G**) Isotretinoin therapy adverse effects—infections, n = 370. (**H**) Isotretinoin therapy adverse effects—mood and neurological changes, n = 370. (**I**) Isotretinoin therapy adverse effects—others, n = 370.

(**A**)	
**Adverse Effect**	**Percentage [%] (n)**
Xerosis	70% (259)
Retinoid dermatitis	20% (77)
Hair loss	5.9% (22)
Pruritus (itching)	8.4% (31)
Seborrhea	3.5% (13)
Hand eczema	3.5% (13)
Desquamation	3.25% (12)
Pityriasis capitis	3% (11)
Onycholysis	1.6% (6)
Facial edema	1.1% (4)
Dermatographic urticaria	1.1% (4)
Lichen planus	<1% (3)
Folliculitis decalvans	<1% (2)
Photosensitivity	<1% (2)
Trachyonychia	<1% (2)
Skin maceration	<1% (2)
(**B**)	
**Adverse Effect**	**Percentage [%] (n)**
Cheilitis	15.5% (57)
Glossitis	<1% (1)
(**C**)	
**Adverse Effect**	**Percentage [%] (n)**
Epistaxis	3.7% (14)
Sore throat	1.1% (4)
Sinus pain	<1% (2)
(**D**)	
**Adverse Effect**	**Percentage [%] (n)**
Xerophthalmia	4.3% (16)
Vision changes	<1% (3)
Conjunctivitis	<1% (2)
(**E**)	
**Adverse Effect**	**Percentage [%] (n)**
Arthralgia	6.75% (25)
Myalgia	4.9% (18)
Back pain	3.25% (12)
(**F**)	
**Adverse Effect**	**Percentage [%] (n)**
Abdominal pain	3.7% (14)
Nausea	1.6% (6)
(**G**)	
**Adverse Effect**	**Percentage [%] (n)**
Impetigo contagiosa	<1% (3)
Furunculus	<1% (2)
Herpes simplex	<1% (2)
(**H**)	
**Adverse Event**	**Percentage (%) (n)**
Fatigue, drowsiness	7.8% (29)
Headaches Exacerbation of depression	4.6% (17) 1.4% (5)
Dizziness	1.1% (4)
Emotional lability	<1% (3)
Tremors	<1% (2)
(**I**)	
**Adverse Effect**	**Percentage [%] (n)**
Menstrual disorders	1.6% (6)
Angiogranuloma	<1% (2)
Cherry angioma	<1% (2)
Hematoma	<1% (2)
Polyuria	<1% (2)
Polydipsia	<1% (2)
Petechiae	<1% (1)

**Table 6:** Correlations between adverse effects and clinical variables.

Adverse Effect	Variable	Spearman ρ	*p*-Value
Hand eczema	Daily dose	+0.082	0.0370
Pruritus	Cumulative dose	−0.088	0.0367
Ro-dermatitis	Age	−0.080	0.0286
Desquamation	Age	+0.083	0.0228

**Table 7:** Lipid profile (mg/dL) of the study group—descriptive statistics.

Parameter	Cases	Mean	SD	Median	Min	Max
TC	303	173.9	38.0	169.0	65.0	327.0
HDL	206	58.7	18.0	55.1	28.7	183.0
LDL	195	104.4	33.4	98.0	42.2	227.0
TGs	295	96.3	49.0	82.4	33.0	387.0

TC—total cholesterol; HDL—high-density lipoprotein; LDL—low-density lipoprotein; TGs—triglycerides; Cases—number of observations; SD—standard deviation; Min—minimum value; Max—maximum value.

**Table 8:** Impact of isotretinoin on lipid profile.

Parameter	Cases (+)	Cases (All)	Controls (+)	Controls (All)	OR	OR (95% CI)	RR	RR (95% CI)	*p*-Value
TC	104	303	64	300	1.93	1.34–2.77	1.61	1.23–2.10	0.0004
HDL	80	206	38	300	2.68	1.75–4.10	2.2	1.55–3.12	<0.0001
LDL	105	195	41	300	3.4	2.26–5.10	2.55	1.87–3.47	<0.0001
TGs	51	295	29	300	1.95	1.20–3.17	1.78	1.16–2.72	0.0062
Dyslipidemia	195	303	121	300	2.67	1.92–3.71	1.6	1.36–1.88	<0.0001

**Table 9:** Impact of isotretinoin on aminotransferases.

Parameter	Cases (+)	Cases (All)	Controls (+)	Controls (All)	OR	OR (95% CI)	RR	RR (95% CI)	*p*-Value
AST	17	310	5	200	2.26	0.82–6.23	2.19	0.82–5.84	0.1
ALT	20	309	13	200	1.51	0.73–3.12	1.46	0.75–2.85	0.27

**Table 10:** Impact of isotretinoin on thyroid-stimulating hormone.

Parameter	Cases (+)	Cases (All)	Controls (+)	Controls (All)	OR	OR (95% CI)	RR	RR (95% CI)	*p*-Value
TSH	17	227	10	200	1.54	0.69–3.45	1.5	0.70–3.20	0.29

**Table 11:** Impact of isotretinoin on prolactin.

Parameter	Cases (+)	Cases (All)	Controls (+)	Controls (All)	OR	OR (95% CI)	RR	RR (95% CI)	*p*-Value
PRL	39	127	5	100	8.42	2.97–23.84	6.14	2.51–15.05	<0.00001

**Table 12:** Timing of common adverse effects after isotretinoin’s initiation.

Adverse Event	Typical Time to First Onset	Clinical Note
Cheilitis	within 7 days	Usually the earliest signal of intolerance.
Xerosis	2–3 weeks	Dose-dependent; emollient prophylaxis recommended.
Epistaxis	by month 1	Often follows nasal dryness; topical ointment useful for prevention.
Xerophthalmia	6 weeks–3 months	Managed with lubricating drops; contact lens intolerance should be considered.
Dyslipidemia	by week 4	Detected on laboratory testing.
Musculoskeletal pain	2–3 months	Usually mild.

## Data Availability

The data presented in this study were collected and recorded in a manner that ensures anonymity. Data are available from the corresponding author upon request. The data are not publicly available due to privacy and ethical restrictions.

## Abbreviations

The following abbreviations are used in this manuscript:

ACTH	adrenocorticotropic hormone
ALP	alkaline phosphatase
ALT	alanine aminotransferase
APL	acute promyelocytic leukemia
AR	androgen receptor
AST	aspartate aminotransferase
ATG7	autophagy-related gene 7
ATRA	all-trans retinoic acid
BIRC5	baculoviral inhibitor of apoptosis repeat-containing 5
BMI	body mass index
CASP1	caspase 1
CBC	complete blood count
CDKN1A	cyclin-dependent kinase inhibitor 1A
CI	confidence interval
CML	chronic myelogenous leukemia
CRP	C-reactive protein
CTCL	cutaneous T-cell lymphoma
DR4/DR5	death receptor 4/5
EMA	European Medicines Agency
fT3	free triiodothyronine
fT4	free thyroxine
FDA	Food and Drug Administration
FC	fecal calprotectin
FOXO1/FOXO3	forkhead box O1/O3 protein
GGTP	gamma-glutamyl transpeptidase
GH	growth hormone
HDL	high-density lipoprotein
IBD	inflammatory bowel disease
IGF-1	insulin-like growth factor 1
IGF1R	insulin-like growth factor 1 receptor
IQR	interquartile range
ISO	isotretinoin
KA	keratoacanthoma
LDL	low-density lipoprotein
LH	luteinizing hormone
MF/SS	mycosis fungoides/Sézary syndrome
NHL	non-Hodgkin lymphoma
OR	odds ratio
PEG	polyethylene glycol
PCD	programmed cell death
PPP	Pregnancy Prevention Program
PRL	prolactin
RAR	retinoic acid receptor
RARE	retinoic acid response element
RR	relative risk
RXR	retinoid X receptor
SCC	squamous cell carcinoma
SD	standard deviation
SmPC	summary of product characteristics
SREBF1	sterol regulatory element-binding transcription factor 1
TC	total cholesterol
TG/TGs	triglyceride(s)
TRAIL	TNF-related apoptosis-inducing ligand
TSH	thyroid-stimulating hormone
ρ	Spearman correlation coefficient
